# 4D ultrasound‐based strain assessment of cardiac dysfunction in male rats with reperfused and nonreperfused myocardial infarction

**DOI:** 10.14814/phy2.16159

**Published:** 2024-07-22

**Authors:** Ana C. M. Omoto, Conner C. Earl, Alyssa M. Richards, Karthik Annamalai, Benjamin Nelson, John E. Hall, Craig J. Goergen, Alexandre A. da Silva

**Affiliations:** ^1^ Department of Physiology and Biophysics University of Mississippi Medical Center Jackson Mississippi USA; ^2^ Weldon School of Biomedical Engineering Purdue University West Lafayette Indiana USA

**Keywords:** coronary artery, heart, ischemia–reperfusion, permanent ligation, strain

## Abstract

Two‐dimensional ultrasound (2DUS) echocardiography is the main noninvasive method used to evaluate cardiac function in animal models of myocardial infarction (MI). However, 2DUS echocardiography does not capture regional differences in cardiac contractility since it relies on planar images to estimate left ventricular (LV) geometry and global function. Thus, the current study was designed to evaluate the efficacy of a newly developed 4‐dimensional ultrasound (4DUS) method in detecting cardiac functional differences between two models of MI, permanent ligation (PL), and ischemia/reperfusion (I/R) in rats. We found that only 4DUS was able to detect LV global functional differences between the two models and that 4DUS‐derived surface area strain accurately detected infarcted regions within the myocardium that correlated well with histological infarct size analysis. We also found that 4DUS‐derived strain, which includes circumferential, longitudinal, and surface area strain, correlated with the peak positive of the first derivative of left ventricular pressure (+*dP*/*dt*_max_). In conclusion, 4DUS strain echocardiography effectively assesses myocardial mechanics following experimentally induced ischemia in rats and accurately estimates infarct size as early as 1 day after injury. 4DUS also correlates well with +*dP*/*dt*_max_, a widely used marker of cardiac contractility.

## Graphical abstract

4D ultrasound accurately detects differences in cardiac function in rat models of myocardial infarction and correlates well with histology and ventricular pressure. *E*_a_: surface strain area; 4D/2DUS: 4D/2D Ultrasound; +*dP*/*dt*_max_: peak positive of the first derivative of left ventricle pressure.

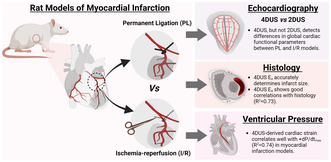

**New and noteworthy**

Our study indicates that unlike 2DUS echocardiography, 4DUS can detect differences in cardiac systolic function between two widely used myocardial infarction models, permanent ligation and ischemia/reperfusion of the left anterior descending coronary artery. We also showed that 4DUS surface area strain accurately estimates infarct size as early as 24 h postsurgery and is a useful tool for longitudinal studies. Finally, our study demonstrated that 4DUS strain correlates well with +*dP*/*dt*_max_, an invasive method that is widely used to assess cardiac contractility.

## 1 INTRODUCTION

Myocardial infarction (MI) is one of the main causes of death worldwide (Khan et al., 2020) with timely reperfusion using stents or thrombolytic drugs being the primary therapeutic intervention for acute coronary artery obstruction (Bahit et al., 2018). In the United States, approximately 25% of patients who suffer an acute MI do not receive timely coronary reperfusion therapy often due to a lack of early medical assistance (Lindsey, de Castro Brás, et al., 2021). Unfortunately, even patients who undergo timely reperfusion interventions can develop myocardium damage, a process known as ischemia/reperfusion (I/R) injury. Although MI with or without reperfusion can lead to left ventricle (LV) dysfunction, there are differences between reperfused and nonreperfused hearts regarding their pathophysiology, remodeling processes, and subsequent progression to LV dysfunction (Lindsey, de Castro Brás, et al., 2021). Distinction between these two MI models, I/R and permanent ligation (PL) of the left anterior descending coronary artery (LAD), is of fundamental importance when evaluating new therapeutic strategies to prevent or attenuate cardiac dysfunction following myocardial infarction (Lindsey, Brunt, et al., 2021).

Two‐dimensional ultrasound (2DUS) echocardiography is often used as a noninvasive method to evaluate LV function and monitor disease progression in these models. However, 2DUS may not completely characterize the complex myocardial biomechanics involved in the pathologic remodeling of the heart since this technique relies on planar images to estimate LV global function. Recently, advances in four‐dimensional ultrasound imaging (4DUS) have allowed for a more comprehensive analysis of LV function in mice in vivo (Damen, Newton, et al., 2021). 4DUS echocardiography provides gated volumetric data of the entire LV with a high spatiotemporal resolution (Damen et al., 2017, 2023; Damen, Newton, et al., 2021; Damen, Salvas, et al., 2021; Dann et al., 2022; Rutledge et al., 2020; Soepriatna et al., 2019); therefore, 4DUS echocardiography may provide regional identification of distinct patterns of contractility within the heart after I/R or PL, enhancing experimental data rigor and reproducibility compared to 2DUS. However, comparisons of 4DUS with 2DUD methods for evaluating cardiac function have not been previously reported in rats, which are widely used to investigate the pathophysiology and treatment of MI.

In the present study, we used two distinct rat models of MI, I/R and PL, to evaluate global and regional cardiac function using 2DUS and 4DUS echocardiography‐based strain analyses at baseline, 24 h, and 7 days after cardiac injury. We assessed potential differences in global and regional cardiac function between the I/R and PL MI models at different time points and compared 2DUS and 4DUS approaches in the evaluation of disease progression. We also examined the potential predictive value of 4DUS surface strain to accurately determine infarct size in rats, which if proven successful would indicate that 4DUS surface strain could be used as a noninvasive method to assess cardiac remodeling over time. Additionally, we analyzed if 2DUS‐ and 4DUS‐derived strain parameters correlate with the peak positive of the first derivative of LV pressure (+*dP*/*dt*_max_) and could be used as a noninvasive method to estimate LV contractility in these ischemic models.

## 2 METHODS

All experimental protocols and procedures were approved by the Institutional Animal Care and Use Committee (IACUC) of the University of Mississippi Medical Center, Jackson, Mississippi (protocol 2022‐1207).

### 2.1 Animals

We performed experiments in male (*n* = 23) 12 to 14‐week‐old Sprague–Dawley rats purchased from ENVIGO (Harlan/Envigo, USA). We placed rats in a 12‐h dark and light cycle and gave all animals free access to food (Teklad 8640) and water throughout the study.

### 2.2 Animal surgeries

#### 2.2.1 Myocardial ischemia–reperfusion and permanent left coronary artery ligation

Rats were anesthetized with isoflurane (3%–5% induction and 2% maintenance) and submitted to endotracheal intubation using polyethylene size 90 tubing. Then, the endotracheal tube was connected to a mechanical ventilator (Harvard Apparatus, USA) at 80 breaths per minute with a tidal volume of 1.2 mL/kg. After steady breathing was established, we opened the chest at the 4th left intercostal space using a chest retractor between the ribs to allow better assessment of the heart. For ligation, the pericardium was removed, and then the left anterior descending coronary artery (LAB) was permanently ligated (PL) the left anterior descending coronary artery (LAD) using a 4–0 prolene suture (Ethicon, USA), or momentarily occluded for 60 min (I/R). Then, the chest retractor was removed, and the ribs were drawn together; thoracic pressure was reestablished, and the skin was sutured. A thin layer of antibiotics (penicillin G benzathine, 1,200,000 U/mL, Pfizer) was applied on the chest before suture to avoid infections. Buprenorphine (0.1 mg/kg, sc) was administered immediately after surgery and 24 h following postoperative recovery.

### 2.3 Echocardiography

#### 2.3.1 Two‐dimensional echocardiography (2DUS)

Transthoracic echocardiography was performed at baseline (before I/R or PL) and on days 1 and 7 after I/R or PL surgeries using a Vevo 3100 ultrasound system (FUJIFILM VisualSonics, Canada) equipped with a high‐frequency transducer with a 21 MHz center frequency (MX250; 15–30 MHz) at 100 frames/s. Rats were anesthetized with 2.5% isoflurane and placed in a supine position on a heating table. Their extremities were fixed to four electrocardiography leads on the table. After the removal of all hair in the chest area, warm Aquasonic 100 gel (Parker Laboratories, USA) was applied to optimize the visibility of the heart chambers. A parasternal long axis B‐mode at the maximum LV length was acquired.

LV ejection fraction (EF) and stroke volume (SV) were calculated as EF = [(LVEDV − LVESV)/LVEDV] × 100 and SV = LVEDV − LVESV, respectively. To calculate cardiac output, stroke volume was multiplied by heart rate. Global longitudinal, circumferential, and radial strain and strain rate were analyzed by 2DUS echocardiography speckle‐tracking using VevoStrain software (FUJIFILM VisualSonics, Canada).

#### 2.3.2 Four‐dimensional echocardiography (4DUS)

4D images were also collected at baseline, day 1, and day 7 after surgery using the same Vevo 3100 System, a 21‐MHz transducer (MX250) and a translating linear step motor. Serial short‐axis ECG‐gated cine loops were acquired with ~11 μm steps across the full LV to construct 4D datasets. Then, the raw 4DUS images were used to quantify cardiac function and tissue strain using a custom‐built graphical user interface (MATLAB, MathWorks, USA), referred to here as the 4D Strain Toolbox (Damen et al., 2023; Damen, Newton, et al., 2021; Damen, Salvas, et al., 2021).

#### 2.3.3 4DUS strain analysis

LV strain was quantified from the acquired 4D images using the 4D Strain Toolbox. First, the ultrasound image was oriented to center in the SAX, LAX, and coronal views of the left ventricle. The apical and basal boundaries were set at peak systole and end diastole and tracked throughout the cardiac cycle. Based on these parameters, initial contours along the epicardial and endocardial boundaries were approximated using a modified ellipsoid prior to manual tracking. Then, the ventricle was segmented by identifying four equally spaced SAX slices each with 3 rotational degrees in the LAX with adjustable points on the endocardium and epicardium. We then use a semi‐manual 4DUS tracking feature method enhanced by machine learning (Damen, Newton, et al., 2021). This segmentation amounts to tracking 48 points on the endocardial and epicardial surfaces over a representative cardiac cycle. After the initial placement of the points by machine learning, the user adjusts the position of each point as appropriate at different frames in a representative cardiac cycle. Automatic interpolation between adjustments ensures adequate temporal tracking. The 4D mesh generated through these tracked points is sampled uniformly at 60 interpolated time points across one cardiac cycle, with 60 rotations around the longitudinal axis, and 60 slices from base to apex for a total of 3600 nodes at each timepoint (Damen et al., 2017). First, circumferential strain (*E*_cc_) was estimated by calculating the circumferential component of the Green Lagrange strain tensor from the 4D mesh at each SAX slice location as follows (Damen, Salvas, et al., 2021; Morrison et al., 2009):

$$E_{\mathrm{cc}}\left(z,t\right)=\frac{1}{2}\left[{\left(\frac{C\left(z,t\right)}{C_{D}\left(z\right)}\right)}^{2}-1\right]\cdot 100\%$$

Here *C* is the circumference of the endocardium in each representative short axis slice oriented orthogonally along the longitudinal direction z over time t during a representative cardiac cycle. *C*_D_ is the circumference at end diastole where t = 0. This produces a strain curve over the cardiac cycle for each SAX slice from which we extracted strain at peak systole as shown previously (Damen, Salvas, et al., 2021). Global peak *E*_cc_ was calculated by averaging strain at peak systole from each slice from apex to base along the *z*‐axis.

Longitudinal strain (*E*_ll_) was estimated using the engineering small strain approximation in the Lagrangian reference frame as follows:

$$E_{\mathrm{ll}}\left(\theta ,t\right)=\left[\frac{L\left(\theta ,t\right)-L_{D}\left(\theta \right)}{L_{D}\left(\theta \right)}\right]\cdot 100\%$$

where L represents the length from apex to base along the circumferential boundary at a rotation θ at time t within the cardiac cycle. $L_{D}$ represents the length at end‐diastole. Localized strain at peak systole was derived from strain curves corresponding to the anterior free wall, anterior, anterior septum, posterior septum, posterior, and posterior free wall sections (Damen, Salvas, et al., 2021). Global peak *E*_ll_ was calculated by taking the average of each region at peak systole.

Finally, surface area strain (*E*_a_) was estimated by calculating it as:

$$E_{a}\left(z,\theta ,t\right)=\left[\frac{A\left(z,\theta ,t\right)-A_{D}\left(z,\theta \right)}{A_{D}\left(z,\theta \right)}\right]\cdot 100\%$$

where A represents the surface area on the endocardial surface between two sequential slice locations along the longitudinal axis z and rotational location θ over time t in the cardiac cycle. Infarct size was estimated by identifying nodes with low *E*_a_ magnitude (<20%) as previously described (Dann et al., 2022). Localized *E*_a_ peak strain was determined by calculating an average metric from within regions defined by the American Heart Association 17‐segment model of the left ventricle (Cerqueira et al., 2002). For strain rate determination, we estimated the slope of the strain curve for each component in systole as described previously (Earl et al., 2023; Leyba et al., 2023).

### 2.4 Ventricular catheterization

For ventricular catheterization, rats were anesthetized with urethane (1 mg/kg) and placed on a temperature‐controlled heating pad to maintain body temperature. Then, a pressure–volume catheter (Millar 1.4F, SPR 838, Millar Instruments, USA) was inserted into the LV through the right carotid artery and the peak positive of the first derivative of LV pressure (+*dP*/*dt*_max_) was calculated during the isovolumetric contractility phase of the cardiac cycle. After LV pressure measurements, rats were euthanized by isoflurane overdose followed by thoracotomy and the removal of the heart for histological analysis.

### 2.5 Histological analysis and infarct size quantification

The hearts were sliced transversely from the apex to the basal part of the LV in four equally spaced cross sections. Then, each cross‐section was embedded in paraffin, sectioned at 5 μm every 300–600 μm, and stained with picrosirius red (PSR) (Abcam, USA – cat# ab246832). Slides with stained cross‐sections were captured using light microscopy (Bio Tek Lionheart FX, Agilent, USA) at 4× magnification. Infarcted tissue was identified as loss of myofibers with replacement by collagen (red dark areas) in PSR‐stained slides and infarct size was calculated by dividing the length of the infarcted area (midline length approach) by the total circumference of the LV (expressed as a percentage) for transmural infarcts (Nascimento et al., 2011). We measured nontransmural infarcts using Bio Tek Gen5 Software (Agilent, USA) to detect noninfarcted and infarcted LV areas based on differences in color. In this case, the infarct size was calculated as the ratio of the infarcted area to the total LV area.

### 2.6 Statistical methods

The results were expressed as means ± SD. Data were tested for normality using the D'Agostino‐Pearson, Shapiro–Wilk, and Kolmogorov–Smirnov tests. Datasets that failed all three tests were analyzed using nonparametric statistics. For normally distributed data, we used a 1‐way ANOVA with repeated measures followed by the Tukey–Kramer post hoc test for comparisons between each group or a 2‐way ANOVA followed by the Tukey–Kramer post hoc test for comparisons between different groups. Single‐time points between two groups were compared using a Student's *t*‐test. Statistical significance was accepted at a level of *p* < 0.05.

## 3 RESULTS

### 3.1 Infarct size differences in PL and I/R models determined by 4DUS surface area strain (*E*_a_) and histology

**FIGURE 1:**
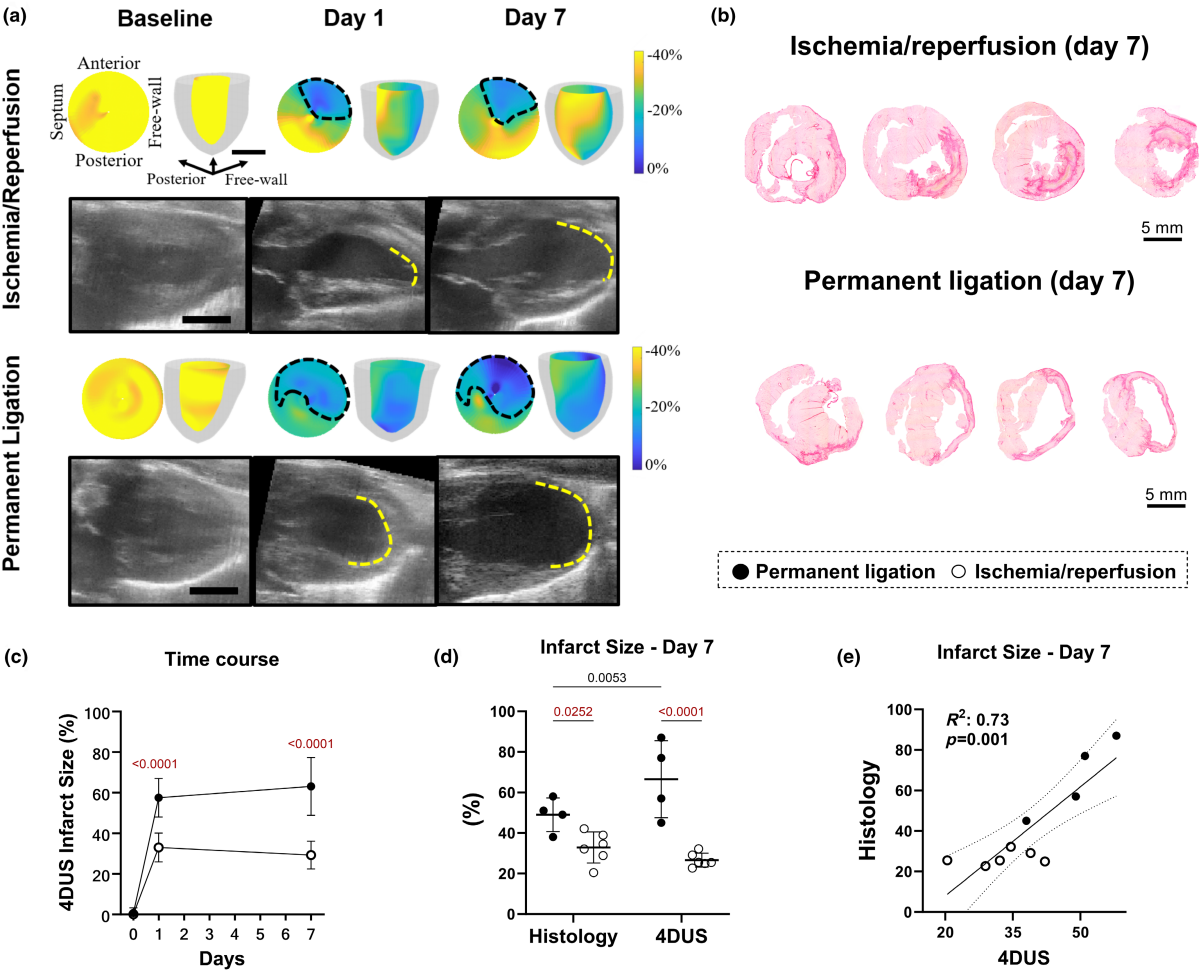
Infarct size differences in PL and IR models determined by 4DUS surface area strain (*E*_a_) and histology. (a) Example of rat left ventricular surface area strain colorized polar plot (left) and 3D model (right) and representative long axis image (below) at baseline, day 1, and day 7 following permanent ligation (PL) or ischemia/reperfusion (I/R). Dotted black lines represent estimated infarct and yellow lines represent areas of akinesis. (b) Representative short‐axis picrosirius red‐stained histological sections at the base, mid‐basal, mid‐apical, and apical regions of the left ventricle (left to right). (c) 4DUS strain‐estimated infarct size over time (PL, *n* = 11; I/R, *n* = 12; day 0 indicates baseline measurements). (d) Comparison between histologically estimated and strain‐estimated infarct size at day 7 postinjury (PL, *n* = 4; I/R, *n* = 6). (e) correlation analysis between strain‐estimated and histologically‐estimated infarct size (PL, *n* = 4; I/R, *n* = 6). Only statistically significant *p* values were showed in the graphs and were highlighted in red for comparisons made between PL and I/R groups. Black scalebar = 5 mm.

We observed significant differences in infarct size (IS) between PL and I/R models with PL resulting in larger IS compared to I/R at day 1, as measured by 4DUS surface area strain (*E*_a_) (Figure 1c), and at day 7, as measured by both 4DUS *E*_a_ and histology (Figure 1d). Additionally, PL caused transmural infarcts, whereas in the I/R model, infarcts were usually not transmural as observed in histological sections where the scar was restricted to the mid‐myocardium, preserving the overall thickness of the ventricular walls (Figure 1b). Despite this consistency in wall thickness, we were able to estimate IS, determined by reductions in peak 4DUS *E*_a_ magnitude (Figure 1a,c). However, in the PL model, where IS was bigger and transmural, 4DUS *E*_a_ slightly overestimated IS when compared to histological measurements at day 7 (Figure 1d). The correlation between histology and 4DUS *E*_a_ at day 7 was positive (*R* ^2^ = 0.73) and significant (*p* = 0.001; Figure 1e). There were no significant differences in 4DUS *E*_a_‐estimated IS between days 1 and 7 postsurgery in both MI models (Figure 1c).

### 3.2 4DUS, but not 2DUS, echocardiography detects differences between PL and I/R models in global cardiac function parameters

**FIGURE 2:**
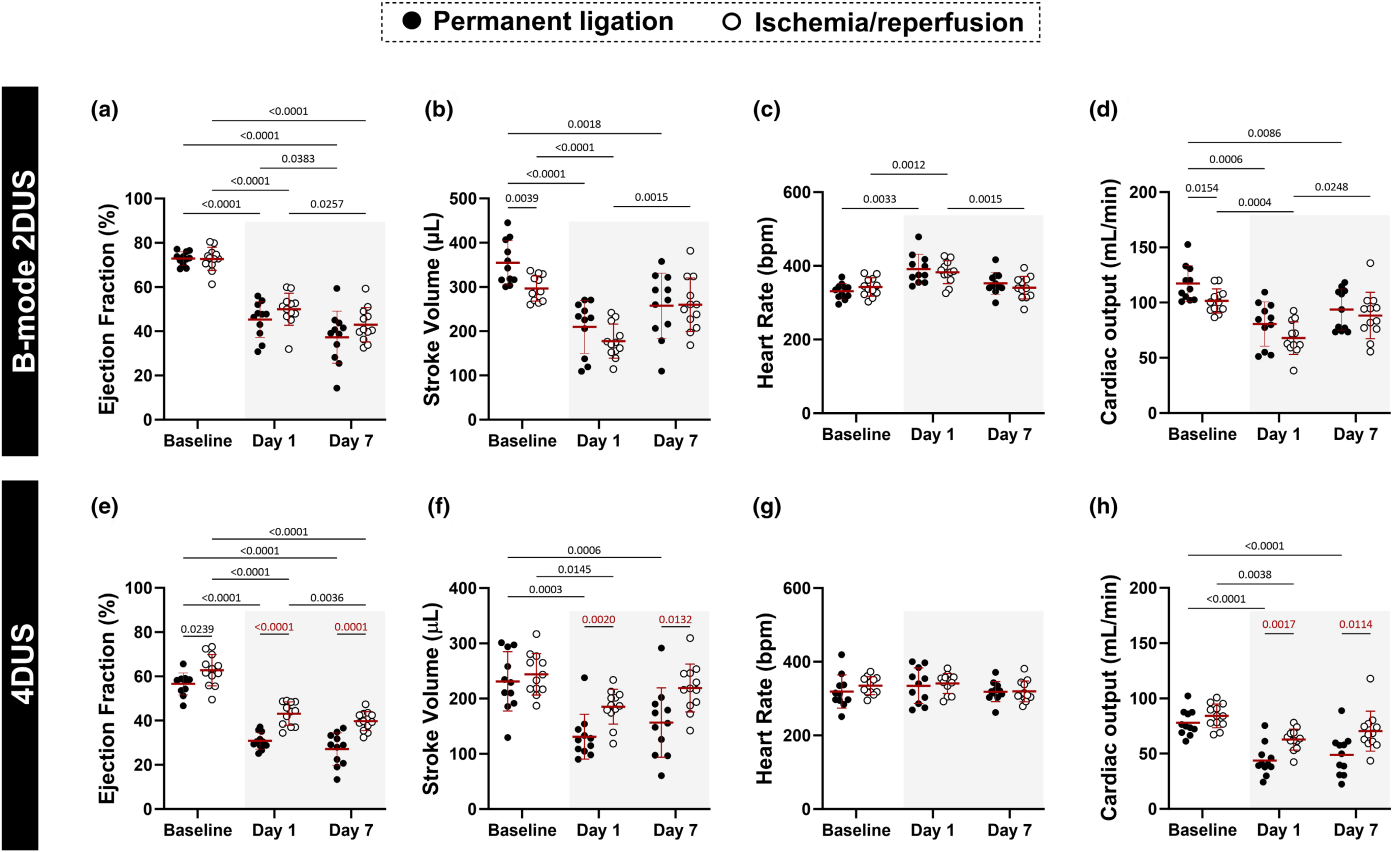
4DUS, but not 2DUS, detects differences between permanent ligation (PL) and ischemia/reperfusion (I/R) in global cardiac function parameters. (a–d) Cardiac parameters estimated between PL (*n* = 11) and I/R (*n* = 12) groups using conventional B‐mode 2DUS analysis (e–h) Cardiac parameters estimated using 4DUS analysis. 2DUS and 4DUS analysis was performed on the same animals. Gray rectangle highlights the days after PL or I/R surgeries. Only statistically significant *p* values were showed in the graphs and were highlighted in red for comparisons made between PL and I/R groups.

As expected, echocardiography parameters of global systolic function such as ejection fraction (EF), stroke volume (SV), and cardiac output (CO) were reduced at days 1 and 7 post PL or I/R procedures compared to baseline (Figure 2a,b,d–f,h), irrespective of ultrasound modality (2DUS or 4DUS). However, while 2DUS did not show differences at either day 1 or 7 when comparing the PL with the I/R model (Figure 2a,b,d), 4DUS was able to detect significant differences in EF, SV, and CO between the MI models, with PL exhibiting larger reductions in these parameters at days 1 and 7 compared to I/R (Figure 2e,f,h). Heart rate (HR) increased from baseline to day 1, and then returned to baseline levels at day 7 when measured by 2DUS (Figure 2c). However, when HR was measured during 4DUS acquisition, no significant differences were observed between time points (Figure 2g). HR did not change significantly between I/R and PL groups regardless of ultrasound modality (Figure 2c,g).

### 3.3 Differences in cardiac strain between PL and I/R models

**FIGURE 3:**
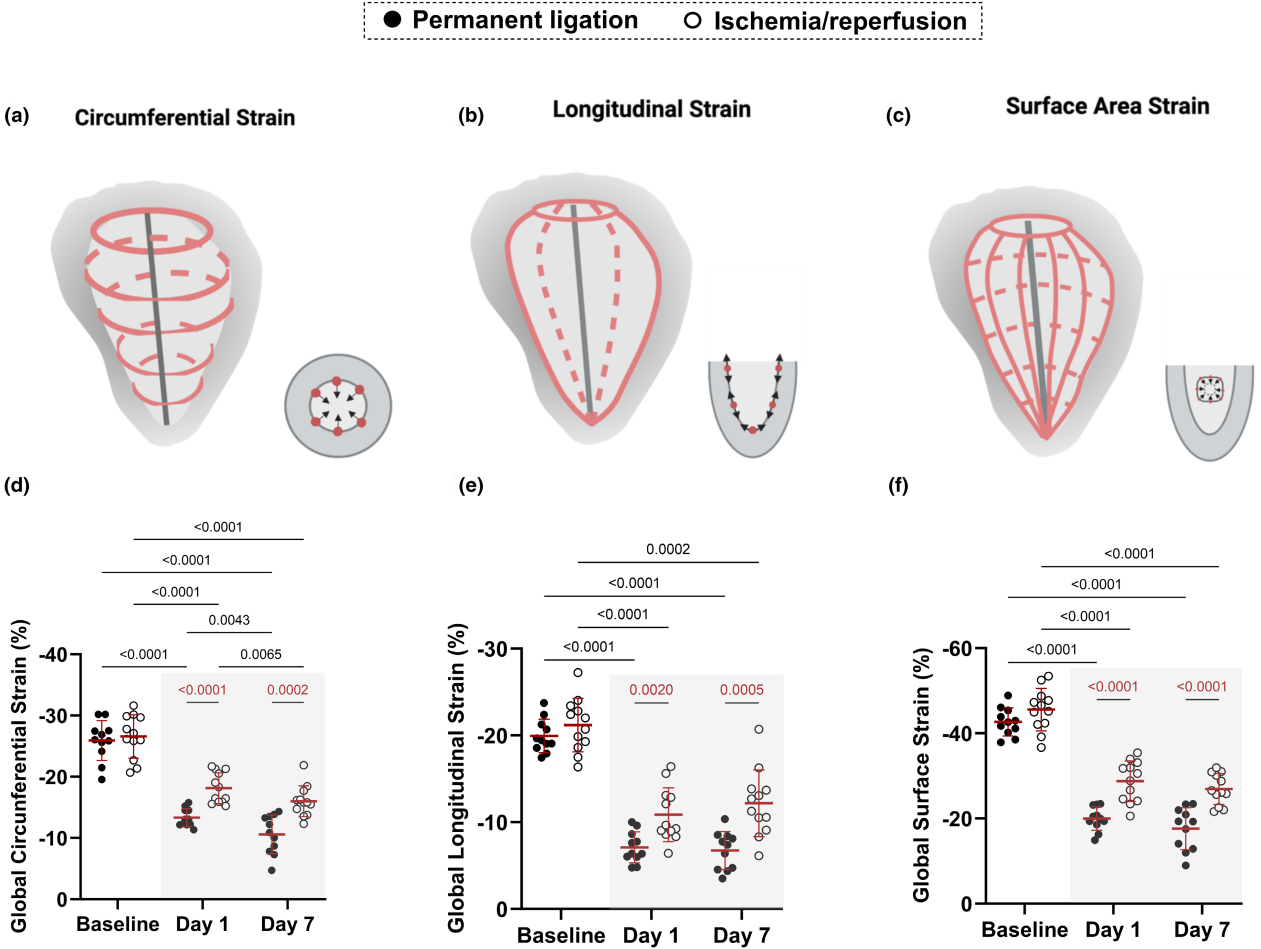
Cardiac fiber strain is differently affected by permanent ligation (PL) and ischemia/reperfusion (I/R) models. (a–c) Example figures showing strain patterns measured by circumferential, longitudinal, and surface area strain. (d–f) Longitudinal characterization of strain differences between PL (*n* = 11) and I/R (*n* = 12) models. Gray rectangle highlights the days after PL or I/R surgeries. Only statistically significant *p* values were showed in the graphs and were highlighted in red for comparisons made between PL and I/R groups.

Directions of myocardial strain are represented in Figure 3a–c. The magnitudes of global *E*_cc_, *E*_ll_, and *E*_a_ using 4DUS were significantly decreased at days 1 and 7 post‐MI when compared to baseline in both PL and I/R groups (Figure 3d–f). In addition, we also observed greater reductions in global *E*_cc_, *E*_ll_, and *E*_a_ in the PL model at days 1 and 7 postsurgery when compared to the I/R model (Figure 3d–f).

### 3.4 4DUS surface area strain reveals distinct patterns of regional cardiac strain in PL and I/R models

**FIGURE 4:**
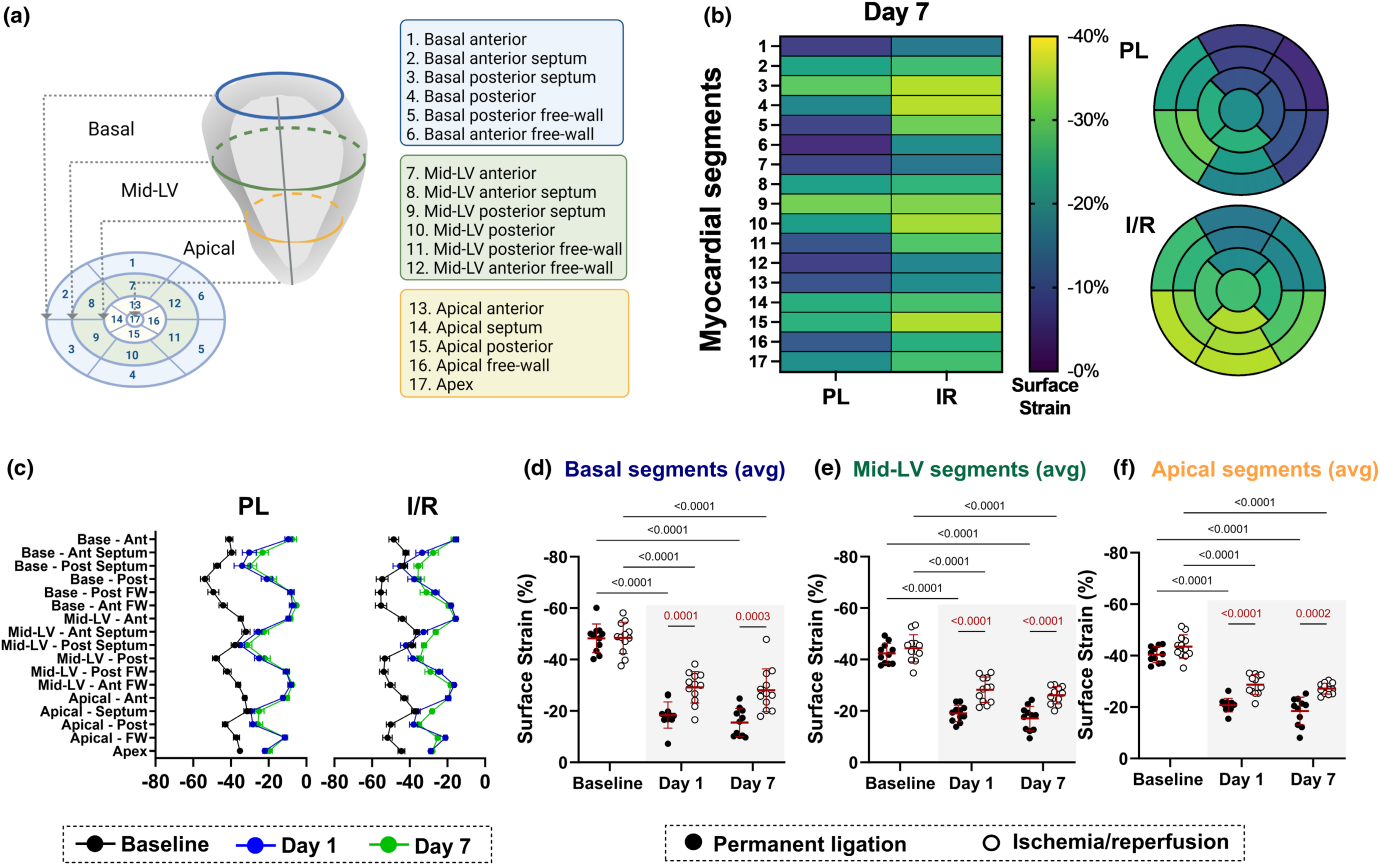
4DUS surface area strain (*E*_a_) reveals distinct patterns of regional contractility in permanent ligation (PL) and ischemia/reperfusion (I/R) models. (a) 17‐segment polar plot map. (b) Regional heat map (left) and polar plots (right) depicting peak systolic *E*_a_ differences between PL and I/R. (c) Graphical depiction of baseline (black line), day 1 (blue line), and day 7 (green line) regional peak *E*_a_ differences for PL and I/R. (d–f) Time course comparison of PL (*n* = 11) and I/R (*n* = 12) in the basal, mid‐LV, and apical peak *E*_a_ averaged segments. Gray rectangle highlights the days after PL or I/R surgeries. Only statistically significant *p* values were showed in the graphs and were highlighted in red for comparisons made between PL and I/R groups.

Regional analysis of 4DUS *E*_a_ (Figure 4a) showed distinct alterations in the patterns of cardiac strain between PL and I/R groups when comparing baseline with days 1 and 7 post surgeries. For instance, PL resulted in overall lower *E*_a_ at day 7 in almost every LV region, but particularly in the anterior and free wall regions of the LV, when compared to I/R (Figure 4b). At baseline (black line), both I/R and PL groups showed a relatively high magnitude of *E*_a_ in the posterior and free wall segments of the LV compared to the anterior and septal regions (Figure 4c). However, at days 1 and 7 post‐MI surgeries (blue and green lines), the largest relative decreases in *E*_a_ occurred in the anterior and free wall portions of the LV along the territory of the myocardium supplied by the left anterior descending coronary artery (Figure 4c). Collectively, PL caused greater reductions in E_a_ magnitude in basal, mid‐LV, and apical regions compared to I/R at days 1 and 7 post‐MI (Figure 4d–f).

### 3.5 4DUS‐derived cardiac strain correlates with +*dP*/*dt*_max_

**FIGURE 5:**
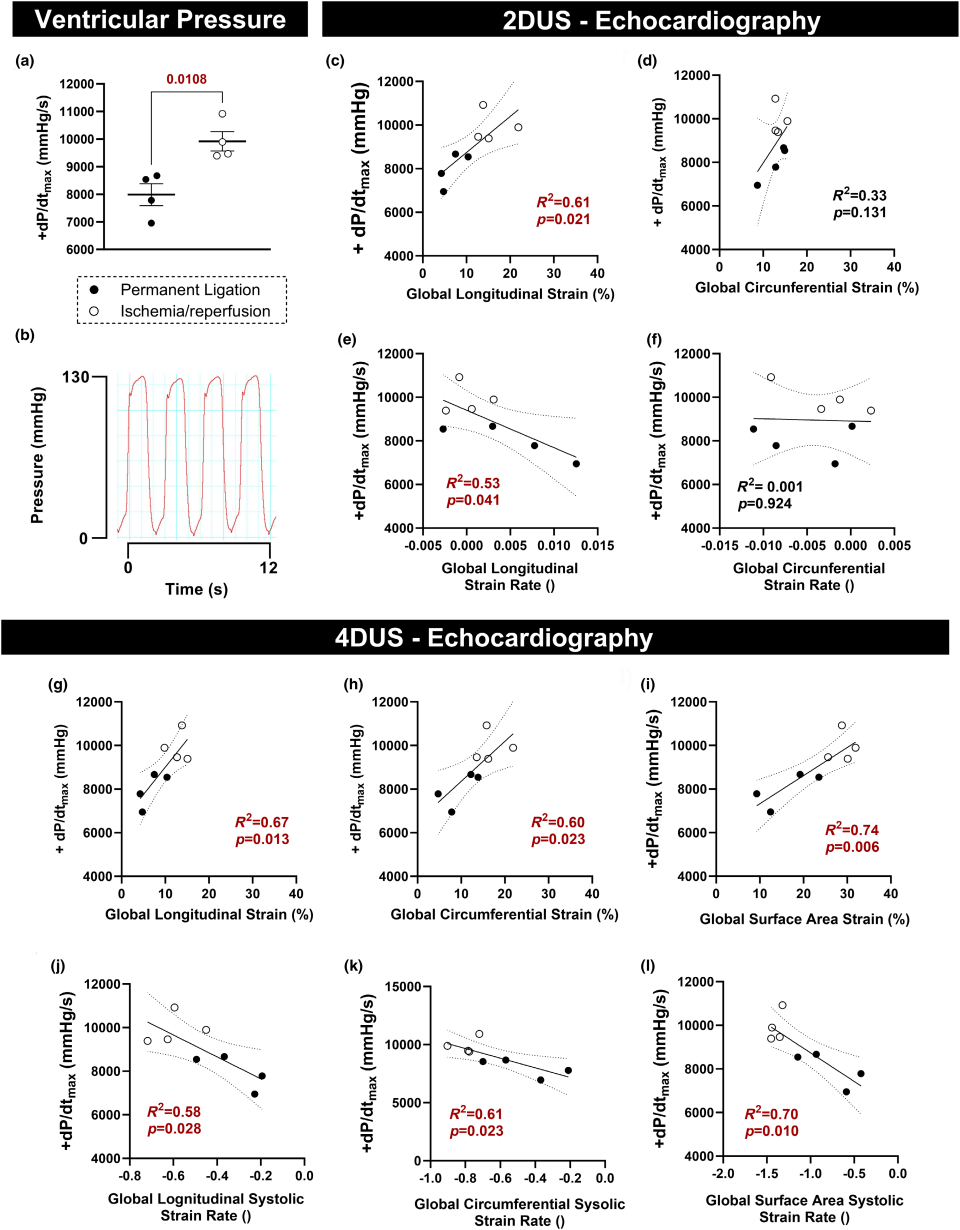
4DUS‐derived strain correlates with +*dP*/*dt*_max_. (a) Peak positive of the first derivative of left ventricle pressure (+*dP*/*dt*_max_) measured at day 7 postsurgery in permanent ligation (PL) and ischemia/reperfusion (I/R) models. (b) representative trace of ventricular pressure over time. (c, d) Correlation of 2DUS‐derived strains and +*dP*/*dt*_max_. (e, f) Correlation of 2DUS‐derived strain rates and +*dP*/*dt*_max_. (g–i) Correlation of 4DUS‐derived strains (normalized to cardiac cycle) and +*dP*/*dt*_max_. (j–l) Correlation of 4DUS‐derived strain rates and +*dP*/*dt*_max_. *p* Values were highlighted in red when differences were statistically significant (*p* < 0.05).

Assessment of LV pressure at day 7 postsurgery showed that the PL model caused a greater reduction in the peak positive of the first derivative of LV pressure (+*dP*/*dt*_max_) compared to the I/R model (Figure 5a). We also observed that only 2DUS‐derived longitudinal strain (Figure 5c) and longitudinal strain rate (Figure 5e) correlated significantly with +*dP*/*dt*_max._ In contrast, all 4DUS‐derived strain parameters (*E*_cc_, *E*_ll_, and *E*_a_) significantly correlated with +*dP*/*dt*_max_ (Figure 5g–i). Among all 4DUS‐derived peak strain parameters, *E*_a_ showed the strongest correlation with +*dP*/*dt*_max_ (*p* = 0.006; *R* ^2^ = 0.74; Figure 5i). We noted similar strong correlation between +*dP*/*dt*_max_ and 4DUS‐derived global strain rate parameters (Figure 5j–l) with *E*_a_ systolic strain rate showing the strongest correlation with +*dP*/*dt*_max_ (*p* = 0.010; *R* ^2^ = 0.70; Figure 5l).

## 4 DISCUSSION

One important and common consideration when using MI animal models to study heart failure is the ability to determine infarct size in vivo using noninvasive methods. Although cardiac magnetic resonance imaging (MRI) and positron emission tomography (PET) have been developed for small rodents (Bohl et al., 2009; Fischer et al., 2021), these methods are expensive and difficult to employ for longitudinal assessment studies. Recent advances in 4DUS echocardiography, however, have made noninvasive longitudinal assessment of infarct size in mice both feasible and reliable, even with complex morphological changes (Dann et al., 2022). Here we apply these techniques in rat models of MI, which are common and widely used in basic research, strengthening the field's ability for longitudinal cardiac remodeling assessment in protocols that aim to reduce infarct size and improve cardiac function.

We also show how our strain estimation method correlates well with histological quantification of infarct size in both PL and I/R MI models. We then correlated our strain metrics with +*dP*/*dt*_max_ to assess cardiac contractility. To test our method, we used two models of MI‐induced heart failure, the I/R approach, which recapitulates the cardiac remodeling that occurs following percutaneous coronary intervention (De Villiers & Riley, 2020), and the PL model which better represents the scar formation and cardiac dysfunction observed in untreated MI (Lindsey, de Castro Brás, et al., 2021).

### 4.1 4DUS *E*_a_ accurately determines infarct size

Our results showed that infarct size measured using *E*_a_ estimated from 4DUS echocardiography at 24 h following an ischemic event accurately predicts infarct size at day 7 postsurgery. This finding is highly impactful to the field because traditional wall‐thinning or histological metrics in rodents can only assess infarct size after significant remodeling and collagen deposition have occurred (Dann et al., 2022; Soepriatna et al., 2019). Using our strain‐estimated infarct size technique, we also observed different impacts of PL versus I/R on cardiac injury, with the PL model resulting in larger infarcts compared to I/R (Figure 1c,d). Additionally, we showed a strong correlation (*R* ^2^ = 0.73) between infarct size measured by the 4DUS strain at day 7 postsurgery and the gold standard histological method (Figure 1e).

It should be noted that our 4DUS strain estimation technique relies on gross akinesis of the myocardium to estimate infarct size, which may be prone to potential overestimation of large infarcts when compared to histology, as 4DUS strain estimation of infarcts may include some portions of the “at‐risk” border zone. This may explain the small, but not significant, decrease in infarct size estimated using the 4DUS strain in I/R rats when comparing day 1 and day 7 postsurgery as the at‐risk border zone may have become more “kinetic” from day 1 to day 7. It is important to highlight that 4DUS infarct size estimation at day 1 is not a measurement of the 24‐h remodeling process of the myocardium (scar formation); instead it is a measurement of the akinetic areas that can predict infarct size at day 7. This can explain why the infarct size on day 1 is statistically similar to that on day 7 post‐I/R or PL (Figure 1c).

Our 4DUS strain method may also underestimates small to mild infarcts that do not induce damage through the entire thickness of the myocardium wall, allowing some layers to retain some amount of contractility. Although there is some variability when comparing infarct size from areas with low surface strain (<20%) measured by 4DUS to histologic infarct size, there is a strong correlation between the values (*R* ^2^ = 0.72) making it a valuable tool for early and longitudinal tracking of infarct size.

### 4.2 4DUS echocardiography distinguishes PL and I/R differences in cardiac function

For decades, traditional B‐mode or M‐mode modalities have been the primary noninvasive techniques for assessing cardiac function. One important limitation of these two methods is that volumetric calculations are based on a 2D video or linear images of the heart chambers and can vary considerably depending on the angle of acquisition when positioning the ultrasound probe. 4DUS echocardiography, on the other hand, is angle‐independent and does not rely on assumptions for volumetric estimations from 2D or linear parameters. This advantage of the 4DUS method is of particular benefit in the context of coronary ischemia as cardiac remodeling often leads to asymmetric remodeling of the ventricles, further complicating angle‐dependent acquisition and estimation of metrics that require formulas designed for normal‐shaped hearts.

For example, we found significant differences in ejection fraction, stroke volume, and cardiac output between PL and I/R models on days 1 and 7 post‐MI surgery using our 4DUS method. In contrast, when using standard B‐mode assessment of the same cardiac parameters, we observed no significant differences between PL and I/R at either time postsurgery. These findings suggest that although B‐mode and 4DUS approaches detected important changes in cardiac function after PL or I/R procedures when compared to baseline, only 4DUS echocardiography could differentiate the impact of PL versus I/R on cardiac function metrics. Additionally, while we did not anticipate differences between PL and I/R groups at baseline, we found small but significant differences in stroke volume at baseline when measured using B‐mode, but not when using our 4DUS approach. One potential explanation for this could be the inherently higher variability in measurements using B‐mode 2DUS compared to 4DUS. Additionally, it should be noted that there were small differences in heart rates between 2DUS and 4DUS acquisitions. This may be due, in part, to the longer acquisition time required for 4D image acquisition compared to 2DUS and thus the longer exposure time to anesthesia. Despite this, we still observed significant differences between I/R and PL groups in 4DUS derived‐stroke volume and cardiac output on day 1 and day 7. Collectively, these studies indicate higher accuracy of measured variables using 4DUS, which shows better correlation with gold standard cardiac MRI (Damen et al., 2017; Damen, Salvas, et al., 2021; Dann et al., 2022; Rutledge et al., 2020).

### 4.3 4DUS strain analysis: A sensitive indicator of cardiac myocardial damage

The orientation and pattern of contraction of cardiac fibers play a critical role in the overall function of the heart. In the setting of an ischemic injury, the health of these fibers and patterns of contraction are significantly disrupted within the territory of the affected muscles. One method to examine the integrity of these patterns of contraction and their disruption is through the analysis of myocardial strain. The changing pattern of strain in ischemic injury may have the potential to provide an early indication of the extent of overall myocardial injury and remodeling that will occur (Dann et al., 2022).

In our 4DUS analysis, we found that *E*_cc_, *E*_ll_, and *E*_a_ components of strain revealed strong differences between PL and I/R groups (Figure 3), demonstrating that 4DUS strain analysis is a sensitive indicator of the extent of myocardial damage, even as early as 24 h following an ischemic injury. This is meaningful as the process of remodeling following an ischemic insult takes time and the ability to accurately quantify early indicators of progression is of great value in both preclinical studies and potentially in the clinical care of patients with MI.

There are many techniques to assess/estimate myocardial strain using ultrasound, with each technique possessing advantages and limitations. Some techniques rely on tracking the speckle pattern and estimating the deformation between timeframes in order to calculate the strain tensor (Voigt et al., 2015). However, this technique can be complicated using B‐mode images since out‐of‐plane motion can skew results. The technique we applied in the present study uses a semi‐manual 4DUS tracking feature method enhanced by machine learning (Damen, Newton, et al., 2021). After estimating the deformation of the myocardium fibers, we generated a full 3D mesh of the endo and epicardium muscles and how they contract along the cardiac cycle. From this, we derived each component of the strain tensor at any region of the myocardium we chose. Because we track features in three dimensions, we can also account for complex motion of the myocardium that cannot be tracked via 2D analysis alone.

### 4.4 Surface area strain (*E*_a_) determines regional differences between PL and I/R models

We found that *E*_a_ was particularly useful in differentiating the impact of PL versus I/R on myocardium kinetics in our analysis. The calculation of *E*_a_ requires measuring the change in size of the endocardial surface of the LV, which is only possible when using 3D images of the left ventricle. It is also unique in that it captures both circumferential and longitudinal shortening in one composite value. *E*_a_ has been used previously with 3D speckle tracking to evaluate cardiomyopathy (Earl et al., 2023; Yu et al., 2019), myocardial infarction outcomes in humans (Shin et al., 2016), animal models of myocardial ischemia (Kozuma et al., 2019), and chemotherapy‐induced cardiotoxicity (Coutinho Cruz et al., 2020; Jasaityte et al., 2013).

Using 4DUS, we showed strong qualitative and quantitative differences in regional patterns of *E*_a_ between PL and I/R groups at days 1 and 7 postinjury. We noted that both I/R and PL groups showed a decrease in peak magnitude *E*_a_ in anterior and free wall segments, and this decrease was more profound in the PL group. Also important were the remarkable changes in the *E*_a_ pattern at days 1 and 7 when compared to baseline, reflecting very prominently the reduced myocardium kinetics within the regions supplied by the LAD, particularly the anterior and free wall segments, while the septal and posterior segments remain relatively unchanged. These regions of low *E*_a_ magnitude (<20%) also correlated well with histological regions of infarct size.

### 4.5 4DUS‐derived strain components correlate with the peak positive of the first derivative of left ventricle pressure (+*dP*/*dt*_max_)

LV contractility is a major determinant of cardiac performance and impaired contractility is an important hallmark of MI‐induced heart failure (Cecconi et al., 2014). Most previous studies using rodent MI models rely on 2DUS echocardiography measurements, especially EF, to estimate cardiac contractility (Monge Garcia et al., 2018). Thus, a reduction in EF is often considered a surrogate for decreased LV contractility. However, EF is a load‐dependent measurement and does not directly reflect the inotropic state of the myocardium (Marwick, 2018). Therefore, measurements of peak positive of the first derivative of LV pressure (+*dP*/*dt*_max_) during the isovolumetric contraction phase of the cardiac cycle may be a better surrogate of cardiac contractility since it is much less affected by changes in preload and afterload. One limitation of this method, however, is that +*dP*/*dt*_max_ measurement requires the insertion of a catheter equipped with a pressure sensor inside the LV which is not feasible for studies that require consecutive longitudinal measurements of cardiac contractility over many days or even months. Thus, a noninvasive echocardiographic parameter that correlates well with +*dP*/*dt*_max_ would be of great value for a more accurate estimation of cardiac contractility in longitudinal studies.

Myocardial strain is considered an index of myocardial fiber deformation along the cardiac cycle and is directly related to LV contractility (Greenberg et al., 2002). In addition, 2DUS strain and strain rate correlate with LV + *dP*/*dt*_max_ in a porcine model of acute dynamic unload produced by vena cava occlusions (Dahle et al., 2016). In this work, we determined how well 2DUS‐ and 4DUS‐derived strain parameters correlate with +*dP*/*dt*_max_ in our MI models and examined whether 4DUS‐derived strain provides better estimation of LV contractility as determined by +*dP*/*dt*_max_. We observed that all measured 4DUS strain parameters (*E*_cc_, *E*_ll_, and *E*_a_) exhibited good and significant correlation with +*dP*/*dt*_max_ and that peak *E*_a_ and *E*_a_ systolic strain rate showing the strongest correlation with +*dP*/*dt*_max_ (*R* ^2^ = 0.74, *R* ^2^ = 0.70). Of the 2DUS strain parameters, only longitudinal strain and longitudinal strain rate showed a significant correlation with +*dP*/*dt*_max_. Thus, 4DUS echocardiography not only distinguished differences between PL and I/R models regarding cardiac function but also provided better noninvasive estimation of LV contractility in these ischemic models. Given the nature of this as an early feasibility study, we note here that these results were obtained with a subset of our rats (PL, *n* = 4; I/R, *n* = 4) and future analysis with more animals may be warranted.

### 4.6 Limitations

Despite the implementation of preliminary tracking algorithms, our current 4DUS strain analysis method excludes valvular geometry and still requires manual correction, particularly when image artifacts (i.e. rib shadow, sternum artifact) are present. This is particularly true when tracking the basal portion of the heart near the mitral valve. Efforts are being made to improve on the automation of our 4DUS tracking in three dimensions, and we anticipate that an improvement in automation will continue to reduce the current analysis time of 30–60 min per dataset to complete a high‐fidelity strain assessment of a rat heart by the user.

## 5 CONCLUSION

We demonstrated that 4DUS strain echocardiography is an effective and useful tool for examining myocardial mechanics following experimentally induced ischemia in rats, one of the most commonly used species in biomedical research. We also found that 4DUS can be used to accurately assess infarct size as early as 1 day after injury. Finally, our study demonstrated that 4DUS strain correlates well with +*dP*/*dt*_max_, an invasive method that is widely used to assess cardiac contractility.

## AUTHOR CONTRIBUTIONS

A.C.M.O., A.A.S. and C.C.E. conceived and designed research; A.C.M.O., C.C.E., A.R., K.A. and B.N. analyzed the data; A.C.M.O., C.C.E., A.A.S. and C.J.G. interpreted results of the experiments; A.C.M.O. and C.C.E. prepared figures; A.C.M.O. and C.C.E. drafted the manuscript; A.C.M.O., C.C.E., A.R., K.A., B.N., J.E.H., C.J.G. and A.A.S. edited and revised the manuscript; A.C.M.O., C.C.E., A.R., K.A., B.N., J.E.H., C.J.G. and A.A.S. approved final version of the manuscript.

## FUNDING INFORMATION

Research reported in this publication was supported by the National Heart, Lung, and Blood Institute of the National Institutes of Health F30HL162452 and 1R01HL163076, the National Institute of General Medical Sciences of the National Institutes of Health P20GM104357, P30GM149404 and U54GM115428, and the American Heart Association POST835218. The content is solely the responsibility of the authors and does not necessarily represent the official views of the National Institutes of Health or the American Heart Association.

## CONFLICT OF INTEREST STATEMENT

C.J.G. is a paid consultant of FUJIFILM VisualSonics.

## ETHICS STATEMENT

The authors take full responsibility for every aspect of this work, ensuring that any questions regarding the accuracy or integrity of any part are thoroughly investigated and addressed.

## Data Availability

The data underlying this article will be shared on reasonable request to the corresponding author.
